# The R.M.O. and the County Court—III

**Published:** 1908-04-04

**Authors:** 


### Resident Medical Officers' Department.

### THE R.M.O. AND THE COUNTY COURT.—III.

### EVIDENCE AND CROSS-EXAMINATION.

When the house officer reaches the witness box and has been duly sworn, his evidence-in-chief is obtained by the counsel for the litigant at whose instance he has been subpœnaed. This begins in a stereotyped way with a summary of professional qualifications, experience, appointments, and so forth. Here the R.M.O. sometimes finds himself at a disadvantage; for though he may be, both by his relation to the case and by reason of his hospital experience, best qualified to give the Court a correct view of the medical aspect of the case, juries, who naturally do not understand that fact, are apt to prefer the evidence of some grey-headed practitioner, on whose forty years' experience counsel for the other side dwells with considerable unction. If, beyond the handicap of age, the house officer, through nervousness, should betray any symptoms of doubt or hesitation, his evidence may be rejected by the jury and some other quite mistaken diagnosis or prognosis accepted.

This leads to the extremely important question of the way to give evidence. It is one on which advice, and very good advice, is offered freely in text-books of medical jurisprudence; but it is one on which the most persistent reiteration is not out of place. The medical witness, whether in a County Court, or any other judicial chamber, should stand well up in the box, and answer all questions with a clear voice. There is no need to shout, for a distinct enunciation should ensure that everyone in court can hear. There is no harm—on the contrary, there may be much advantage—in considering a few seconds before answering each or any question. That will not be construed as hesitancy, but merely as carefulness, if the answer, when it does come, is straightforward and to the point. It is the halting or the unintelligible, not the deliberate, answer which damages a witness's credibility in the eyes of the Court.

More important even than this is the simplicity of the witness's language. He must remember always that the jury are far and away more ignorant of medical technicalities than he is himself of the various crafts and sciences which he has never studied. He must describe everything in the simplest and most every-day language, taking not even the most elementary facts of anatomy or physiology for granted. Thus, the jury do not know that there are two bones in the leg, much less their names; to allude, therefore, to a fracture of the tibia in those terms is merely to cause confusion. Speak of the membrane (perhaps better even, the lining) between the ribs and the lung if you will, but never of the pleura; of the breastbone, not of the sternum, and so on. Even here there are pitfalls, for in country districts especially there are certain folk-names for various structures, whose exact significance the doctor must know if he would use them. Thus, before a Yorkshire jury it would be wiser to stick to the technical "tendons" than to betray ignorance of the distinction between the "leaders" and the "guiders" of the fingers. Freedom from technicalities is the first essential to the making of what lawyers call a good witness: we offer no apology for dwelling upon it at length, because it is the cardinal error over which practically everyone stumbles at first, and which many medical men never seem able to surmount.

Next to clear speaking, and to the avoidance of technical terms, both of which involve of necessity clear thinking, sincerity is the quality of most importance. The expert witness should never betray bias towards either side: he should answer the questions of his cross-examiner with the same frankness as he displays in his examination-in-chief. To take up a semi-hostile, reluctant attitude under ordinary cross-examination is merely to create an impression of undue collusion with the friendly counsel. We speak of ordinary cross-examination to distinguish it from a severely hostile one. As a matter of fact, cross-examination in County Courts is almost always very lenient; for the barrister is, generally speaking, too much afraid of getting beyond his depth to cross-examine a doctor very far. The best way of avoiding this much-dreaded ordeal is to be clear, simple, accurate, and emphatic in the evidence-in-chief: irresolution, technicalities, and hesitancy are so many direct provocations to the opposing barrister. In courts of wider jurisdiction, where larger issues are at stake and more eminent counsel are therefore employed, this immunity from cross-examination cannot so confidently be expected; but the line indicated is in all cases the best for the medical witness to adopt.

The value of systematic register-keeping and note-taking in hospitals is hardly ever more appreciated by the R.M.O. than when, months after some accident, he is called into court to give evidence about its victim. In the stress of a busy hospital it is not easy to remember the exact details of a fracture or concussion case unless careful notes of the case have been taken. Especially important is it to ascertain and commit to writing the state of the patient with regard to alcohol, for the question of sobriety or otherwise is almost certain to be raised in court. A few years ago a woman was treated in a large hospital for very severe injuries sustained when a 'bus ran over her; those injuries necessitated amputation through the thigh. No claim for compensation was made at the time, but the case was afterwards taken up on her behalf, and three years ago application was made at the hospital for details of the injuries. It so happened that in the interval the resident under whose care she had been had died; but on looking up the registers, the top line of the notes, in his handwriting, was found to be, "Patient dead drunk on admission." The case, of course, went no further, and the moral of it needs no pointing.

